# Consistency of trial reporting between ClinicalTrials.gov and corresponding publications: one decade after FDAAA

**DOI:** 10.1186/s13063-020-04603-9

**Published:** 2020-07-23

**Authors:** Ramtin Talebi, Rita F. Redberg, Joseph S. Ross

**Affiliations:** 1 Section of Cardiovascular Medicine, Department of Medicine, University of California at San Francisco School of Medicine, San Francisco, CA USA; 2 Section of General Medicine, Department of Medicine, Yale School of Medicine, New Haven, CT USA; 3 Department of Health Policy and Management, Yale School of Public Health, New Haven, CT USA; 4 Center for Outcomes Research and Evaluation, Yale–New Haven Hospital, New Haven, CT USA

**Keywords:** FDA Amendments Act, Clinical trial reporting, Result reporting, Accurate reporting

## Abstract

The FDA Amendments Act (FDAAA) required that information for certain clinical trials, such as details about study design features and endpoints, as well as results, be publicly reported in ClinicalTrials.gov. We conducted a cross-sectional analysis of phase III trials with primary results published between January 1, 2016, and June 30, 2017, in high-impact journals and found 74% contained at least one discrepancy between results reported in ClinicalTrials.gov and the corresponding publication. Our findings underscore the necessity for monitoring of clinical trial information and result reporting between sources; a checklist may provide a systemized procedure for investigators and editors to monitor accurate reporting.

## Background/aims

The *2007 Food and Drug Administration Amendments Act* (FDAAA) [1] required that information for clinical trials, such as details about study sponsors, design features, sample eligibility criteria, and study endpoints, as well as study results, be publicly reported on ClinicalTrials.gov, a clinical trial registry managed by the National Library of Medicine. Previous studies identified inconsistencies between trial information and the results reported in ClinicalTrials.gov and their corresponding publications [2, 3]. A decade after these initial studies, we sought to characterize the consistency of information and result reporting between ClinicalTrials.gov and corresponding publications for trials published in high-impact journals.

## Methods

Using PubMed, we identified all phase III clinical trials with primary results published between January 1, 2016, and June 30, 2017, in journals with a 2016 Journal Impact Factor of 10 or greater (Supplementary Table 1), linked to an NCTID, and with results reported in ClinicalTrials.gov(Supplementary Figure; list provided in Supplementary Table 2). For all trials, we compared the information and the results reported in the publication and in ClinicalTrials.gov for the following study features: cohort characteristics (completion rate, age, sex, race/ethnicity), intervention details, primary efficacy endpoints, and serious adverse events. These four study features were examined because they were (1) objectively comparable between the two sources and (2), in our estimation, the most important when weighing the design, significance, and interpretation of a trial.

For cohort characteristics, information was deemed concordant if the type of properties reported and the values for each were the same between sources. For intervention details, information was deemed concordant when the dosage, time course, and frequency of the intervention matched. For primary efficacy endpoints, information was deemed concordant when the measure description and the reported results matched. For serious adverse events, information was deemed concordant when the event description and reported results matched. Study features (cohort characteristics, trial intervention details, primary efficacy endpoint, serious adverse events) could not be compared between the two sources when they were reported in different formats. For example, if the age distribution was reported as the number of participants in certain age ranges (18–30, 31–45, ...) rather than as the mean age, or if adverse events were stratified in one source as serious vs. non-serious while in another were reported in aggregate. We conducted a cross-sectional analysis, characterizing the rate of reporting and consistency in the information and results reported for all study features between the two sources using descriptive statistics; all analyses were performed using Excel (version 16.24) and RStudio (version 1.1.447).

## Results

There were 94 phase III clinical trials published in high-impact journals that had results reported on ClinicalTrials.gov; 89 (95%) were funded by industry, 4 (4%) were funded by government institutions, and 1 (1%) was funded by other academic/nonprofit institutions. Trials were most commonly published in *The New England Journal of Medicine* (*n* = 28; 30%), *Annals of the Rheumatic Diseases* (*n* = 14; 15%), and *Lancet Oncology* (*n* = 14; 15%).

Among the 94 trials, the cohort characteristics of completion rate, age, and sex were reported in both sources for 95 to 100% of trials, whereas race/ethnicity was reported in both sources for 35 (37%) trials (Table 1). For trials where completion rate, age, or sex were reported in both sources, these characteristics could not be compared or were discordant for 24–26%. For trials where race/ethnicity was reported in both sources, the race/ethnic distribution could not be compared or was discordant for 12 (34%). Intervention details were reported in both sources for 94 (100%) trials but could not be compared or were discordant for 11 (12%).

**Table 1:** Concordance of result information between ClinicalTrials.gov and corresponding publications for phase III trials published in high-impact journals between January 1, 2016, and June 30, 2017

Result information^**b,c**^	Trials reporting results in both sources, no. (%)	Result information in both sources		
		Concordant, no. (%)	Discordant, no. (%)	Cannot be compared, no. (%)
Cohort characteristics				
Completion rate	94 (100)	69 (73)	22 (23)	3 (3)
Age distribution	93 (99)	70 (75)	20 (22)	3 (3)
Sex distribution	89 (95)	67 (75)	19 (21)	3 (3)
Race/ethnicity distribution	35 (37)	23 (66)	12 (34)	0
Trial intervention	94 (100)	83 (88)	11 (12)	0
Primary efficacy endpoint^a^	94 (100)	70 (74)	20 (21)	4 (4)
Serious adverse events	93 (99)	41 (44)	24 (26)	28 (30)

^a^Total number of primary efficacy endpoints reported on ClinicalTrials.gov = 128 compared to publications = 112

^b^Total percentage of trials with at least one discrepancy across all categories = 87%

^c^Total percentage of trials with at least one discrepancy across all categories excluding race = 74%

Primary efficacy endpoints were reported by both sources for 94 (100%) trials, of which 4 (4%) could not be compared and 20 (21%) were discordant. Among these 20 discordant studies, 1 had a different endpoint reported between the two sources and 19 had different results reported between the two sources for the same endpoint. Serious adverse events were reported by both sources for 93 (99%) trials, of which 28 (30%) could not be compared and 24 (26%) were discordant. Serious adverse event results could most often not be compared because the two sources reported their results in different formats or stratifications. For example, the publication might report serious adverse events broken down by type, while ClinicalTrials.gov would report an aggregate number, or vice versa. Overall, excluding race/ethnicity, 74% of trials had at least one discrepancy in information and result reporting (discordant or could not be compared) between ClinicalTrials.gov and corresponding publications across all study features.

## Conclusion

A decade after the FDAAA, 74% of phase III trials published in high-impact journals contained some discrepancy in the information and results reported in ClinicalTrials.gov and their corresponding publication, marking only a slight decrease from studies of a decade prior [2, 3]. Such high rates of discordance suggest that the challenges of providing clear and consistent trial information and reported results across public sources of information still need to be addressed. Most concerning were the inconsistencies observed in reporting of primary efficacy endpoint results and serious adverse events. While the magnitude of these discrepancies was often small, such as small differences in cohort characteristics (e.g., in reported mean age), these discrepancies raise questions as to which source is correct. Potential explanations include that the publications may have reported on a differently defined cohort than the original trial or that trials were published before additional study observations accrued or after statistical analyses were refined and ClinicalTrials.gov was not subsequently updated. Despite inconsistencies between registered and published primary outcomes of clinical trials being recently observed among a broad sample of clinical trials [4], our findings are particularly concerning because we focused on phase III trials published in high-impact journals, which are the trials that likely have the greatest influence on clinical care and are used in clinical practice guidelines.

Our study was limited to an 18-month sample of phase III trials that were registered and reported results in both ClinicalTrials.gov and published in high-impact journals. Thus, it is likely that our study examined those trials following the best practices with respect to result reporting, making our estimates of discrepancies in reporting conservative. Investigators and sponsors that are reporting results to ClinicalTrials.gov, in addition to publishing their study in the highest impact journals, are more likely to adhere to best practices as compared to those who fail to report results to ClinicalTrials.gov. Nevertheless, these findings underscore the necessity for monitoring for concordance of clinical trial information and results reported between these sources. We propose a three-pronged approach to ensuring a harmonious reporting of result information: a checklist for investigators to use to ensure congruent reporting pre-submission to a journal, an acknowledgement of any differences that investigators recognize in the submitted manuscript, and a post-submission check by the journal editors. Sponsors and investigators face several challenges to accurate and consistent result reporting, including a high rate of research staff turnover, lack of staff dedicated to monitoring result reporting at many academic institutions and smaller companies, and poor knowledge of FDA and NIH reporting requirements. A checklist—similar to those applied in surgical settings—may provide a systemized procedure for investigators to monitor accurate reporting to ClinicalTrials.gov throughout the trial process. Additionally, investigators that recognize differences between the results in their manuscript and those in ClinicalTrials.gov should explain these in the study publication. And finally, journal editors, upon receiving a submission, should request that the trial sponsors provide a link to the corresponding ClinicalTrials.gov entry and an itemized list of consistencies between the most important trial features (such as the four we examine in this study).

## Supplementary information

**Additional file 1: Table S1.** Alphabetized list of journals with a 2016 Journal Impact Factor of 10 or greater. **Table S2.** List of articles reporting the primary results of Phase III clinical trials published between January 1st, 2016 and June 30th, 2017 in journals with a 2016 Journal Impact Factor of 10 or greater, linked to an NCTID, and with results reported in ClinicalTrials.gov. **Figure S1.** Sample Cohort Flow Diagram.

## Data Availability

The datasets generated and/or analyzed used for the current study were derived from PubMed and the *ClinicalTrials.gov* repository, https://clinicaltrials.gov/. The list of journals is provided in Supplementary Table 1, and the list of articles is provided in Supplementary Table 2.

## Abbreviations

FDAAA: The Food and Drug Administration Amendments Act
