# Supplementary material for: Consistency of trial reporting between ClinicalTrials.gov and corresponding publications: one decade after FDAAA
Source: Trials. 2020 Jul 23;21:675. doi: 10.1186/s13063-020-04603-9 (PMC7376878; doi:10.1186/s13063-020-04603-9)
Supplement: Supplementary file 1 — Additional file 1: Table S1. Alphabetized list of journals with a 2016 Journal Impact Factor of 10 or greater. Table S2. List of articles reporting the primary results of Phase III clinical trials published between January 1st, 2016 and June 30th, 2017 in journals with a 2016 Journal Impact Factor of 10 or greater, linked to an NCTID, and with results reported in ClinicalTrials.gov. Figure S1. Sample Cohort Flow Diagram. [file 13063_2020_4603_MOESM1_ESM.docx]

**SUPPLEMENTARY MATERIALS**

**TRLS-D-20-00072R2**

***“Consistency of Trial Reporting Between ClinicalTrials.gov and Corresponding Publications: One Decade after FDAAA”***

**Table 1:** Alphabetized list of journals with a 2016 Journal Impact Factor of 10 or greater.

- Accounts Of Chemical Research
- ACS Nano
- Acta Neuropathologica
- Advanced Drug Delivery Reviews
- Advanced Energy Materials
- Advanced Functional Materials
- Advanced Materials
- Advances In Optics And Photonics
- Advances In Organometallic Chemistry
- Advances In Physics
- Alzheimers & Dementia
- American Journal Of Gastroenterology
- American Journal Of Human Genetics
- American Journal Of Psychiatry
- American Journal Of Respiratory And Critical Care Medicine
- Angewandte Chemie-international Edition
- Annals Of Internal Medicine
- Annals Of The Rheumatic Diseases
- Annual Review Of Astronomy And Astrophysics
- Annual Review Of Biochemistry
- Annual Review Of Biomedical Engineering
- Annual Review Of Biophysics
- Annual Review Of Cell And Developmental Biology
- Annual Review Of Clinical Psychology
- Annual Review Of Clinical Psychology
- Annual Review Of Condensed Matter Physics
- Annual Review Of Entomology
- Annual Review Of Fluid Mechanics
- Annual Review Of Genetics
- Annual Review Of Immunology
- Annual Review Of Marine Science
- Annual Review Of Materials Research
- Annual Review Of Medicine
- Annual Review Of Microbiology
- Annual Review Of Neuroscience
- Annual Review Of Pathology-mechanisms Of Disease
- Annual Review Of Pharmacology And Toxicology
- Annual Review Of Physical Chemistry
- Annual Review Of Physiology
- Annual Review Of Plant Biology
- Annual Review Of Psychology
- Annual Review Of Public Health
- Applied Physics Reviews
- Astronomy And Astrophysics Review
- Astrophysical Journal Supplement Series
- Behavioral And Brain Sciences
- Biological Psychiatry
- Biological Reviews
- Blood
- BMJ - British Medical Journal
- Brain
- CA - A Cancer Journal For Clinicians
- Cancer Cell
- Cancer Discovery
- Cell
- Cell Host & Microbe
- Cell Metabolism
- Cell Research
- Cell Stem Cell
- Chemical Reviews
- Chemical Society Reviews
- Circulation
- Circulation Research
- Clinical Microbiology Reviews
- Coordination Chemistry Reviews
- Ecology Letters
- Endocrine Reviews
- Energy & Environmental Science
- European Heart Journal
- European Urology
- Fems Microbiology Reviews
- Gastroenterology
- Genes & Development
- Genome Biology
- Genome Research
- Gut
- Hepatology
- Human Reproduction Update
- Immunity
- Intensive Care Medicine
- JAMA
- JAMA Internal Medicine
- JAMA Psychiatry
- Journal Of Allergy And Clinical Immunology
- Journal Of Clinical Investigation
- Journal Of Clinical Oncology
- Journal Of Experimental Medicine
- Journal Of Hepatology
- Journal Of Photochemistry And Photobiology C-photochemistry Reviews
- Journal Of The American Chemical Society
- Journal Of The American College Of Cardiology
- Journal Of The National Cancer Institute
- Lancet
- Lancet Diabetes & Endocrinology
- Lancet Global Health
- Lancet Global Health
- Lancet Infectious Diseases
- Lancet Neurology
- Lancet Oncology
- Lancet Respiratory Medicine
- Leukemia
- Light-science & Applications
- Living Reviews In Relativity
- Living Reviews In Solar Physics
- Materials Science & Engineering R-reports
- Materials Today
- Microbiology And Molecular Biology
- MMWR - Morbidity And Mortality Weekly Report
- Molecular Aspects Of Medicine
- Molecular Biology And Evolution
- Molecular Cell
- Molecular Psychiatry
- Molecular Systems Biology
- Nano Energy
- Nano Letters
- Nano Today
- Natural Product Reports
- Nature
- Nature Biotechnology
- Nature Cell Biology
- Nature Chemical Biology
- Nature Chemistry
- Nature Climate Change
- Nature Communications
- Nature Genetics
- Nature Geoscience
- Nature Immunology
- Nature Materials
- Nature Medicine
- Nature Methods
- Nature Nanotechnology
- Nature Neuroscience
- Nature Photonics
- Nature Physics
- Nature Reviews Cancer
- Nature Reviews Cardiology
- Nature Reviews Clinical Oncology
- Nature Reviews Drug Discovery
- Nature Reviews Endocrinology
- Nature Reviews Gastroenterology & Hepatology
- Nature Reviews Genetics
- Nature Reviews Immunology
- Nature Reviews Microbiology
- Nature Reviews Molecular Cell Biology
- Nature Reviews Neurology
- Nature Reviews Neuroscience
- Nature Reviews Rheumatology
- Nature Structural & Molecular Biology
- Neuron
- New England Journal Of Medicine
- Pharmacological Reviews
- Pharmacology & Therapeutics
- Physics Reports-review Section Of Physics Letters
- Physiological Reviews
- Plos Medicine
- Progress In Energy And Combustion Science
- Progress In Lipid Research
- Progress In Materials Science
- Progress In Neurobiology
- Progress In Polymer Science
- Psychological Bulletin
- Psychological Science In The Public Interest
- Reports On Progress In Physics
- Reviews
- Reviews Of Geophysics
- Reviews Of Modern Physics
- Science
- Science Translational Medicine
- Studies In Mycology
- Trends In Biochemical Sciences
- Trends In Biotechnology
- Trends In Cell Biology
- Trends In Cognitive Sciences
- Trends In Ecology & Evolution
- Trends In Immunology
- Trends In Neurosciences
- Trends In Pharmacological Sciences
- Trends In Plant Science
- Wiley Interdisciplinary Reviews-computational Molecular Science
- World Psychiatry

**Table 2:** List of articles reporting the primary results of Phase III clinical trials published between January 1^st^, 2016 and June 30^th^, 2017 in journals with a 2016 Journal Impact Factor of 10 or greater, linked to an NCTID, and with results reported in ClinicalTrials.gov.

| **Article Title** | **NCT ID** |
| --- | --- |
| Lung Deflation and Cardiovascular Structure and Function in Chronic Obstructive Pulmonary Disease. A Randomized Controlled Trial. | 1691885 |
| The first double-blind, randomised, parallel-group certolizumab pegol study in methotrexate-naive early rheumatoid arthritis patients with poor prognostic factors, C-OPERA, shows inhibition of radiographic progression. | 1451203 |
| Riociguat for the treatment of pulmonary arterial hypertension associated with connective tissue disease: results from PATENT-1 and PATENT-2. | 810693 |
| Value of ultrasonography as a marker of early response to abatacept in patients with rheumatoid arthritis and an inadequate response to methotrexate: results from the APPRAISE study. | 767325 |
| Apremilast, an oral phosphodiesterase 4 inhibitor, in patients with psoriatic arthritis and current skin involvement: a phase III, randomised, controlled trial (PALACE 3). | 1212770 |
| Ixekizumab, an interleukin-17A specific monoclonal antibody, for the treatment of biologic-naive patients with active psoriatic arthritis: results from the 24-week randomised, double-blind, placebo-controlled and active (adalimumab)-controlled period of the phase III trial SPIRIT-P1. | 1695239 |
| Baricitinib in patients with inadequate response or intolerance to conventional synthetic DMARDs: results from the RA-BUILD study. | 1721057 |
| MRI assessment of suppression of structural damage in patients with rheumatoid arthritis receiving rituximab: results from the randomised, placebo-controlled, double-blind RA-SCORE study. | 578305 |
| Tocilizumab in early progressive rheumatoid arthritis: FUNCTION, a randomised controlled trial. | 1007435 |
| Efficacy and safety of subcutaneous tocilizumab versus intravenous tocilizumab in combination with traditional DMARDs in patients with RA at week 97 (SUMMACTA). | 1194414 |
| Efficacy and safety of ustekinumab in psoriatic arthritis patients with peripheral arthritis and physician-reported spondylitis: post-hoc analyses from two phase III, multicentre, double-blind, placebo-controlled studies (PSUMMIT-1/PSUMMIT-2). | 1009086 |
| Efficacy and safety of ustekinumab in psoriatic arthritis patients with peripheral arthritis and physician-reported spondylitis: post-hoc analyses from two phase III, multicentre, double-blind, placebo-controlled studies (PSUMMIT-1/PSUMMIT-2). | 1077362 |
| A phase III randomised, double-blind, parallel-group study comparing SB4 with etanercept reference product in patients with active rheumatoid arthritis despite methotrexate therapy. | 1895309 |
| A randomised, double-blind, phase III study comparing SB2, an infliximab biosimilar, to the infliximab reference product Remicade in patients with moderate to severe rheumatoid arthritis despite methotrexate therapy. | 1931181 |
| Certolizumab pegol in combination with dose-optimised methotrexate in DMARD-naÃ¯ve patients with early, active rheumatoid arthritis with poor prognostic factors: 1-year results from C-EARLY, a randomised, double-blind, placebo-controlled phase III study. | 1519791 |
| Long-acting recombinant coagulation factor IX albumin fusion protein (rIX-FP) in hemophilia B: results of a phase 3 trial. | 1496274 |
| Long-term remissions after FCR chemoimmunotherapy in previously untreated patients with CLL: updated results of the CLL8 trial. | 281918 |
| Phase 3 trial of defibrotide for the treatment of severe veno-occlusive disease and multi-organ failure. | 358501 |
| Panobinostat plus bortezomib and dexamethasone in previously treated multiple myeloma: outcomes by prior treatment. | 1023308 |
| Effectiveness of Elbasvir and Grazoprevir Combination, With or Without Ribavirin, for Treatment-Experienced Patients With Chronic Hepatitis C Infection. | 2105701 |
| Repeat Treatment With Rifaximin Is Safe and Effective in Patients With Diarrhea-Predominant Irritable Bowel Syndrome. | 1543178 |
| Randomized, controlled trial of entecavir versus placebo in children with hepatitis B envelope antigen-positive chronic hepatitis B. | 1079806 |
| Effect of Macitentan on the Development of New Ischemic Digital Ulcers in Patients With Systemic Sclerosis: DUAL-1 and DUAL-2 Randomized Clinical Trials. | 1474109 |
| Effect of Macitentan on the Development of New Ischemic Digital Ulcers in Patients With Systemic Sclerosis: DUAL-1 and DUAL-2 Randomized Clinical Trials. | 1474122 |
| Effect of Etelcalcetide vs Placebo on Serum Parathyroid Hormone in Patients Receiving Hemodialysis With Secondary Hyperparathyroidism: Two Randomized Clinical Trials. | 1788046 |
| Effect of Etelcalcetide vs Cinacalcet on Serum Parathyroid Hormone in Patients Receiving Hemodialysis With Secondary Hyperparathyroidism: A Randomized Clinical Trial. | 1896232 |
| Effect of Insulin Glargine Up-titration vs Insulin Degludec/Liraglutide on Glycated Hemoglobin Levels in Patients With Uncontrolled Type 2 Diabetes: The DUAL V Randomized Clinical Trial. | 1952145 |
| Effect of Naltrexone-Bupropion on Major Adverse Cardiovascular Events in Overweight and Obese Patients With Cardiovascular Risk Factors: A Randomized Clinical Trial. | 1601704 |
| Effect of Abaloparatide vs Placebo on New Vertebral Fractures in Postmenopausal Women With Osteoporosis: A Randomized Clinical Trial. | 1343004 |
| Pan Canadian Rash Trial: A Randomized Phase III Trial Evaluating the Impact of a Prophylactic Skin Treatment Regimen on Epidermal Growth Factor Receptor-Tyrosine Kinase Inhibitor-Induced Skin Toxicities in Patients With Metastatic Lung Cancer. | 473083 |
| A Randomized, Open-Label, Multicenter, Phase III Study of Epoetin Alfa Versus Best Standard of Care in Anemic Patients With Metastatic Breast Cancer Receiving Standard Chemotherapy. | 338286 |
| Efficacy and Safety of Trabectedin or Dacarbazine for Metastatic Liposarcoma or Leiomyosarcoma After Failure of Conventional Chemotherapy: Results of a Phase III Randomized Multicenter Clinical Trial. | 1343277 |
| Molecular Heterogeneity and Response to Neoadjuvant Human Epidermal Growth Factor Receptor 2 Targeting in CALGB 40601, a Randomized Phase III Trial of Paclitaxel Plus Trastuzumab With or Without Lapatinib. | 770809 |
| Lapatinib in Combination With Capecitabine Plus Oxaliplatin in Human Epidermal Growth Factor Receptor 2-Positive Advanced or Metastatic Gastric, Esophageal, or Gastroesophageal Adenocarcinoma: TRIO-013/LOGiC--A Randomized Phase III Trial. | 680901 |
| Adalimumab for prevention of uveitic flare in patients with inactive non-infectious uveitis controlled by corticosteroids (VISUAL II): a multicentre, double-masked, randomised, placebo-controlled phase 3 trial. | 1124838 |
| Romiplostim in children with immune thrombocytopenia: a phase 3, randomised, double-blind, placebo-controlled study. | 1444417 |
| Isavuconazole versus voriconazole for primary treatment of invasive mould disease caused by Aspergillus and other filamentous fungi (SECURE): a phase 3, randomised-controlled, non-inferiority trial. | 412893 |
| Fulvestrant 500 mg versus anastrozole 1 mg for hormone receptor-positive advanced breast cancer (FALCON): an international, randomised, double-blind, phase 3 trial. | 1602380 |
| Benralizumab, an anti-interleukin-5 receptor Î± monoclonal antibody, as add-on treatment for patients with severe, uncontrolled, eosinophilic asthma (CALIMA): a randomised, double-blind, placebo-controlled phase 3 trial. | 1914757 |
| Efficacy and safety of benralizumab for patients with severe asthma uncontrolled with high-dosage inhaled corticosteroids and long-acting Î²2-agonists (SIROCCO): a randomised, multicentre, placebo-controlled phase 3 trial. | 1928771 |
| Regorafenib for patients with hepatocellular carcinoma who progressed on sorafenib treatment (RESORCE): a randomised, double-blind, placebo-controlled, phase 3 trial. | 1774344 |
| Hydroxycarbamide versus chronic transfusion for maintenance of transcranial doppler flow velocities in children with sickle cell anaemia-TCD With Transfusions Changing to Hydroxyurea (TWiTCH): a multicentre, open-label, phase 3, non-inferiority trial. | 1425307 |
| Eribulin versus dacarbazine in previously treated patients with advanced liposarcoma or leiomyosarcoma: a randomised, open-label, multicentre, phase 3 trial. | 1327885 |
| Ibrutinib versus temsirolimus in patients with relapsed or refractory mantle-cell lymphoma: an international, randomised, open-label, phase 3 study. | 1646021 |
| Nanoliposomal irinotecan with fluorouracil and folinic acid in metastatic pancreatic cancer after previous gemcitabine-based therapy (NAPOLI-1): a global, randomised, open-label, phase 3 trial. | 1494506 |
| Adjuvant sunitinib or sorafenib for high-risk, non-metastatic renal-cell carcinoma (ECOG-ACRIN E2805): a double-blind, placebo-controlled, randomised, phase 3 trial. | 326898 |
| Everolimus for the treatment of advanced, non-functional neuroendocrine tumours of the lung or gastrointestinal tract (RADIANT-4): a randomised, placebo-controlled, phase 3 study. | 1524783 |
| Blood pressure and glycaemic effects of dapagliflozin versus placebo in patients with type 2 diabetes on combination antihypertensive therapy: a randomised, double-blind, placebo-controlled, phase 3 study. | 1195662 |
| Switching from tenofovir disoproxil fumarate to tenofovir alafenamide in antiretroviral regimens for virologically suppressed adults with HIV-1 infection: a randomised, active-controlled, multicentre, open-label, phase 3, non-inferiority study. | 1815736 |
| Efficacy, safety, and immunogenicity of the human papillomavirus 16/18 AS04-adjuvanted vaccine in women older than 25 years: 7-year follow-up of the phase 3, double-blind, randomised controlled VIVIANE study. | 294047 |
| Ceftazidime-avibactam or best available therapy in patients with ceftazidime-resistant Enterobacteriaceae and Pseudomonas aeruginosa complicated urinary tract infections or complicated intra-abdominal infections (REPRISE): a randomised, pathogen-directed, phase 3 study. | 1644643 |
| Efficacy, safety, and tolerability of lacosamide monotherapy versus controlled-release carbamazepine in patients with newly diagnosed epilepsy: a phase 3, randomised, double-blind, non-inferiority trial. | 1243177 |
| Carfilzomib and dexamethasone versus bortezomib and dexamethasone for patients with relapsed or refractory multiple myeloma (ENDEAVOR): a randomised, phase 3, open-label, multicentre study. | 1568866 |
| Ponatinib versus imatinib for newly diagnosed chronic myeloid leukaemia: an international, randomised, open-label, phase 3 trial. | 1650805 |
| Standard first-line chemotherapy with or without nintedanib for advanced ovarian cancer (AGO-OVAR 12): a randomised, double-blind, placebo-controlled phase 3 trial. | 1015118 |
| Afatinib plus vinorelbine versus trastuzumab plus vinorelbine in patients with HER2-overexpressing metastatic breast cancer who had progressed on one previous trastuzumab treatment (LUX-Breast 1): an open-label, randomised, phase 3 trial. | 1125566 |
| Quality of life in patients with advanced renal cell carcinoma given nivolumab versus everolimus in CheckMate 025: a randomised, open-label, phase 3 trial. | 1668784 |
| Effects of sodium thiosulfate versus observation on development of cisplatin-induced hearing loss in children with cancer (ACCL0431): a multicentre, randomised, controlled, open-label, phase 3 trial. | 716976 |
| Cabozantinib versus everolimus in advanced renal cell carcinoma (METEOR): final results from a randomised, open-label, phase 3 trial. | 1865747 |
| Tumour-infiltrating lymphocytes in advanced HER2-positive breast cancer treated with pertuzumab or placebo in addition to trastuzumab and docetaxel: a retrospective analysis of the CLEOPATRA study. | 567190 |
| Obinutuzumab plus bendamustine versus bendamustine monotherapy in patients with rituximab-refractory indolent non-Hodgkin lymphoma (GADOLIN): a randomised, controlled, open-label, multicentre, phase 3 trial. | 1059630 |
| Palonosetron versus ondansetron for prevention of chemotherapy-induced nausea and vomiting in paediatric patients with cancer receiving moderately or highly emetogenic chemotherapy: a randomised, phase 3, double-blind, double-dummy, non-inferiority study. | 1442376 |
| Anamorelin in patients with non-small-cell lung cancer and cachexia (ROMANA 1 and ROMANA 2): results from two randomised, double-blind, phase 3 trials. | 1387269 |
| Anamorelin in patients with non-small-cell lung cancer and cachexia (ROMANA 1 and ROMANA 2): results from two randomised, double-blind, phase 3 trials. | 1387282 |
| Cobimetinib combined with vemurafenib in advanced BRAF(V600)-mutant melanoma (coBRIM): updated efficacy results from a randomised, double-blind, phase 3 trial. | 1689519 |
| Fulvestrant plus palbociclib versus fulvestrant plus placebo for treatment of hormone-receptor-positive, HER2-negative metastatic breast cancer that progressed on previous endocrine therapy (PALOMA-3): final analysis of the multicentre, double-blind, phase 3 randomised controlled trial. | 1942135 |
| Adalimumab in Patients with Active Noninfectious Uveitis. | 1138657 |
| Two Phase 3 Trials of Adalimumab for Hidradenitis Suppurativa. | 1468207 |
| Two Phase 3 Trials of Adalimumab for Hidradenitis Suppurativa. | 1468233 |
| Osimertinib or Platinum-Pemetrexed in EGFR T790M-Positive Lung Cancer. | 2151981 |
| Prolonged Survival in Stage III Melanoma with Ipilimumab Adjuvant Therapy. | 636168 |
| Nivolumab for Recurrent Squamous-Cell Carcinoma of the Head and Neck. | 2105636 |
| Phase 3 Trials of Ixekizumab in Moderate-to-Severe Plaque Psoriasis. | 1474512 |
| Phase 3 Trials of Ixekizumab in Moderate-to-Severe Plaque Psoriasis. | 1597245 |
| Phase 3 Trials of Ixekizumab in Moderate-to-Severe Plaque Psoriasis. | 1646177 |
| A Multinational Trial of Prasugrel for Sickle Cell Vaso-Occlusive Events. | 1794000 |
| Ocrelizumab versus Interferon Beta-1a in Relapsing Multiple Sclerosis. | 1247324 |
| Ocrelizumab versus Interferon Beta-1a in Relapsing Multiple Sclerosis. | 1412333 |
| A Placebo-Controlled Trial of Obeticholic Acid in Primary Biliary Cholangitis. | 1473524 |
| Ustekinumab as Induction and Maintenance Therapy for Crohn's Disease. | 1369329 |
| Ustekinumab as Induction and Maintenance Therapy for Crohn's Disease. | 1369342 |
| Ustekinumab as Induction and Maintenance Therapy for Crohn's Disease. | 1369355 |
| Daratumumab, Lenalidomide, and Dexamethasone for Multiple Myeloma. | 2076009 |
| Daratumumab, Bortezomib, and Dexamethasone for Multiple Myeloma. | 2136134 |
| Bezlotoxumab for Prevention of Recurrent Clostridium difficile Infection. | 1241552 |
| Bezlotoxumab for Prevention of Recurrent Clostridium difficile Infection. | 1513239 |
| Pembrolizumab versus Chemotherapy for PD-L1-Positive Non-Small-Cell Lung Cancer. | 2142738 |
| Oral Ixazomib, Lenalidomide, and Dexamethasone for Multiple Myeloma. | 1564537 |
| Ribociclib as First-Line Therapy for HR-Positive, Advanced Breast Cancer. | 1958021 |
| Tofacitinib as Induction and Maintenance Therapy for Ulcerative Colitis. | 1458574 |
| Tofacitinib as Induction and Maintenance Therapy for Ulcerative Colitis. | 1458951 |
| Tofacitinib as Induction and Maintenance Therapy for Ulcerative Colitis. | 1465763 |
| Palbociclib and Letrozole in Advanced Breast Cancer. | 1740427 |
| Efficacy of Recombinant Influenza Vaccine in Adults 50 Years of Age or Older. | 2285998 |

**Supplementary Figure:** Sample Cohort Flow Diagram.

Publications in journals with JIF>10 and linked to an NCTID number (n=8356)

Excluded (n=7167), reported results from Phase I, II, and IV trials

Publications reporting results from Phase III Trials (n=1189)

Published between 01/01/2016 and 06/30/2017 (n=207)

Excluded (n= 982), published before January 1, 2016 or after June 30, 2017

Publications with results posted on ClinicalTrials.gov (n=98)

Final Sample of Trial Publications (n=94)

Excluded (n= 109), trial results were not posted to ClinicalTrials.gov as of 08/01/2017

Excluded (n=4), articles did not report on the primary outcome of trials
